# Physiological Enquiry Respecting the Action of Moxa, in Various Diseases

**Published:** 1827-04

**Authors:** 


*A Physiological Enquiry respecting the Action of Moxa, and its Utility in inveterate Cases of Sciatica, Lumbago, Paraplegia, Epilepsy, and some other painful, paralytic, and spasmodic Diseases of the Nerves and Muscles.* By WILLIAM WALLACE, M.R.I.A. &c. Surgeon to the Charitable Infirmary of Dublin; and to the Infirmary for the Treatment of Rheumatism and Cutaneous Diseases, in that City, Lecturer on Semeiology and Clinical Surgery. One vol. 8vo. pp. 148. Dublin, Hodges and M'Arthur, and W. Curry and Co.; London, Longman and Co.; and M'Lachlan and Stewart, Edinburgh. 1827.

THE author is anxious to overcome the prejudices which exist in these kingdoms against the use of moxa, and desirous to exalt a remedy so valuable in public estimation. He is certain that the employment of it is impeded by its mode of action having been compared and confounded with that of other agents; and he strives to demonstrate, *in limine*, that the prevailing opinions respecting this subject, are altogether erroneous; and to prove that the moxa affords a remedy which cannot be equalled. His views are illustrated by select cases of an unequivocal nature, because other measures had been tried in vain, and the cures were principally accomplished by this agent; and his opportunities have been greater than those of hospital surgeons in general, from a connexion of above eight years with the Dublin Infirmary for Diseases of the Skin.

The moxa may be defined to be any substance whose gradual combustion on the skin is requisite for the relief or cure of disease. The remedy is, in truth, a very ancient one, but its present celebrity on the Continent arises from the writings and exertions of Larrey, Percy, Dupuytren, Pelletan, Richerand, and Roux. The effects of it have been erroneously compared with those of a blister or issue, whereas, when properly applied, and in appropriate cases, the moxa produces neither inflammation nor discharge, except in rare instances, and even then they are contingent, and not salutary occurrences. The moxa has been condemned as a most painful application; yet those that have undergone it have acknowledged that the pain is not equal to that of a blister, or caustic issue. Sir William Temple, a name dear to every Englishman as the patron of Swift, applied the moxa to his own person, and thus describes its consequences. "For the pain of the burning itself, the first time it is sharp, so that a man may be allowed to complain: I resolved I would not, but that I would count to a certain number, as the best measure how long it lasted: I told *six score and four as fast as I could*, and when the fire of the moxa was out, *all pain of burning was over*. The second time was not near so sharp as the first, and the third a great deal less than the second. The wound was not raw, as I expected, but looked only scorched and black."* Kempfer† observes, "The pain is not very considerable, and falls far short of that which is occasioned by other caustics or actual cauteries. I have seen many times the very boys suffer themselves to be burnt in several parts of their body, without shewing the least sense of pain."

The author, after stating certain experiments and observations, concludes that the application of a precise degree of caloric to the body is followed by a *contraction of the capillaries*, and an *increase in the rapidity of their circulation;* and that this contraction does not proceed from a mere *physical* action, but is the result of the influence of heat on the *vital properties* of the vessels. Yet it is important to decide, whether this influence of caloric in exciting the actions of the frame continues beyond the period of its application. Experiments and observations would seem to prove that it does;—but another question springs from this, namely, are the effects of caloric limited to the point to which it is directly applied, or do they extend more or less beyond that point? There is sufficient proof that caloric can excite an action in living structures to a considerable extent; for it acts on the capillaries as a local tonic, or stimulating astringent, and the power which this class of remedies possesses, of extending their influence by sympathy along continuous surfaces, or similar textures, is well known. The direct effects of the moxa are seldom, if ever, confined to the skin. If an eschar be formed, that eschar extends to the superficial fascia, or subcutaneous cellular tissue; and if the moxa be applied, through the medium of a needle, the caloric may be made to propagate its influence to any depth. Caloric, then, applied under certain circumstances, and with appropriate restrictions, stimulates, in a powerful manner, the capillary vessels, causing them to act with more force, to contract their diameters, and to circulate their blood with greater velocity; and, either by this action on the capillaries, or by a direct one on the lymphatics of the part, it has also the power of exciting the function of the absorbents in a remark-

* Letter of Sir W. Temple, Bart. &c. Swift's Ed. p. 135, vol. I. London, 1720.

† History of Japan, vol. II. sect. iv. page 39.

able manner. Again, the beneficial influence of moxa, in relieving or curing disease, apparently depends on its tonic action over absorption and capillary circulation. Hence, it should be employed in those cases only, in which there exists a state of debility of the capillaries, a consequent retardation of their circulation, and a diminution of absorption; and invariably avoided whenever there is increased action, or active inflammation.

"I have heard of, I shall not say irretrievable mischief, from the employment of moxa in the state of increased action and acute inflammation; but I shall say, I have often heard of much unnecessary pain having been produced, and considerable aggravation of those symptoms which it was meant to relieve. I will candidly admit, that misapplication of a similar kind occurred in my own practice, before I had formed a correct opinion respecting the mode of action of this powerful remedy. Having been taught to suppose that its therapeutic influence is analogous to that of a blister, I formerly employed it indiscriminately in those cases in which blisters are commonly applied; and it is not necessary to observe, after what has been said, that it must have often failed to produce the relief I intended." "Although the effects of moxa are so universally compared to those of a blister, it can never be used upon such a principle. Certainly heat may be so applied as to excite, like blisters, vesication and discharge, and cutaneous inflammation; but, while it produces these effects on the skin, it will act on the deep-seated parts as a powerful stimulant, and thereby often cause, in cases to which blisters are peculiarly suited, more injury than it can do good." "Let it, therefore, be laid down as a principle not to be deviated from, that this remedy shall never be employed in cases of increased action, or of active inflammation, or even in cases of subacute inflammation, that is, when the acute inflammation is lapsing into the state of chronic action; and this principle should be implicitly adhered to, whether the active inflammation has attacked parts previously in a state of health, or has supervened on the state of passive inflammation." 49, 50.

If a part be not fit for the application of the moxa, it must be made so by appropriate means. One of the most useful external remedies for the reduction of increased action is the warm bath; yet caution is demanded relative to its temperature. If applied as an antiphlogistic, it should not be used higher than 97 Fahr. nor lower than 80°. The patient should also remain in it, provided no inconvenience be experienced, forty minutes; and, in general, a much longer period. Evaporating lotions are of considerable utility, and, for their perfect and constant application, the author has invented a very convenient instrument; and of which a plate is given. It is now employed in the Charitable Infirmary of Dublin with great advantage; not only because it affords the means of treating certain cases with accuracy and care, but also from its causing a saving of expenditure in bed-linen. Such an instrument, in our opinion, deserves the attention of every surgeon; and is well worthy of immediate trial; and consequent adoption, if it realize expectation. We would observe, that what Mr. Wallace writes on this, and similar subjects, is highly deserving of consideration, inasmuch as he has long been intimately connected with large public establishments, in Dublin, where the moxa, evaporating lotions, cupping, friction, and shampooing, are necessarily used on a most extensive scale, and under the most auspicious circumstances. For these reasons, we shall transcribe his interesting observations on a new mode of cupping; and, ultimately, advert to his remarks on friction, and introduce his account of the practice of shampooing.

"I prefer the following mode of performing the operation of cupping; and, since I have adopted it, I have never failed to obtain any quantity of blood required; nor have I ever seen it fail in the hands of those pupils who have adopted it by my directions. In the first instance, let me observe, that for the purpose of exhausting the cupping-glasses, the combustion of a few drops of any spirituous liquid on a little tow or lint, is the mode which I much prefer; not only because it is more convenient, by enabling us to dispense with a number of instruments, but also because the degree of heat produced renders the circulation in the cutaneous capillaries more rapid; and, consequently, facilitates the discharge of blood when these vessels have been divided. A cupping-glass having been thus exhausted and applied for some minutes to the part, as well for the purpose of determining to the skin, as to mark the extent of surface which should be scarified, with the assistance of a lancet-scarificator or gum lancet, as it has been called, drawn lightly over the skin and with great rapidity, I traverse the surface, as far as the mark formed by the edge of the glass, with superficial incisions about the fourth of an inch distant from one another. These incisions should be so slight as to be scarcely visible. The operator need not fear that they will not bleed, for the moment the cupping-glass shall be applied, the blood will be found to stream from them with a rapidity which quite surprises those, who, for the first time, have seen the operation thus performed. The reader may suppose that it is more tedious, and, therefore, more painful; but, I assure him, he would be surprised at the rapidity with which the lancet-scarificator may be made to traverse the surface; and that this operation is not so painful as that in common use, I have had innumerable opportunities of ascertaining. The structure of the integument explains the superiority of this mode of operating, and affords proper rules for its performance. The vascularity of the skin is so very different on the external and on the internal surface, that the former may be considered as one of the most vascular textures of the body, while the latter is one of the least vascular." 62.

There are various modes of applying the moxa. Different substances have been used in different countries to produce moxabustion. The Nomades employed wool, and certain spongy matters growing upon oaks, and springing from the hazel—the Indian, the pith of the reed, and flax, or hemp, impregnated with some combustible material—the Armenian, the agaric of the oak—the Chinese and Japanese, the down of the artemisia—the Thessalian, dried moss—the Egyptian, Arracanese, and several oriental nations, cotton—the Ostiaks and Laplanders, the agaric of the birch—and the Aborigines of North America, cotton and dried wood. Baron Percy prefers the medulla of the helianthus annuus, or sun flower, and when this cannot be obtained, cotton impregnated with a saturated solution of the nitrate of potash; while Larrey employs a cylinder of carded cotton, unimpregnated, but surrounded by a capsule of linen. Caloric, however, is the only therapeutic agent evolved during the combustion of these various substances, and consequently that should be selected whose combustion is slow and steady. The author forms his moxa, by immersing either surgeon's lint, or fine linen, in a filtered solution of chlorate of potash, in the proportion of one drachm to four ounces of distilled water. This burns slowly, but steadily; even the blow-pipe produces no sparks; and there is neither trouble to the operator, nor alarm to the patient, from the combustion being scarcely observable. Fine linen will answer best for moxas of a small size. The substance used must be perfectly dry when folded, should possess a proper degree of firmness, and after it has been rolled up, and fastened with two or three stitches of a needle, the end ought to be cut to make it level for application. The length of the moxa should be about three-fourths of an inch, and the diameter may vary from one quarter to a whole inch.

"The instruments which I use in applying the moxa are of the most simple kind: a porte-aiguille, which I have invented (see plate), or a pair of dressing or artery forceps, furnished with a screw at about three-fourths of an inch distant from their point, which screw serves to press the blades of the forceps very tightly together; a bit of small, flat, silver wire, about three inches in length; a bit of card paper; a blow-pipe; a set of needles; and a small glass tube, are all that are required. With the silver wire a small hoop is formed to grasp the moxa, the size of the hoop being made to vary according to the size of the moxa; and the ends of the hoop are grasped in the forceps, which are made tight on it by the screw with which they are furnished. The hoop should be applied about a line distant from that end of the moxa which is to be placed on the skin." 68.

The moxa should be applied, in painful affections, to the point where the greatest distress exists; and, in paralytic diseases, over the origin of the nerves which lead to the morbid parts, and afterwards along the same nerves in different positions of their course. There is scarcely a part in the body to which it may not be applied in one form or other; even to the eye, in the form of the objective moxa, as has been often done by our author, with great advantage, in obstinate chronic ophthalmia. But we must specially attend to the size of the moxa on every occasion, the manner as well as the length of its application, and these must be regulated by the depth of the disease, and the nature of the textures. It may be used so as not to cause any injury; in a greater degree, so as to produce vesication; and, in a still greater degree, an eschar, and the eschar may be either deep or superficial; or, finally, it may be employed with the acupuncture needle. These modes may be denominated the *first*, *second*, *third*, *fourth*, and *fifth* forms of application of the moxa. The *first* will answer when the disease is very superficial; it is the objective cautery of the French authors, and is deemed a powerful remedy for the cure of ulcers. It may be serviceable in neuralgia, when the nerve is superficially situated; or in affections of the joints, where the synovial membrane is, as in the knee and wrist, immediately under the integuments. The moxa should be repeated at least once a day, and applied by holding it, within the forceps, as close to the part as is endurable. The *second* is seldom used by Mr. Wallace, because less effectual than the *third*, and more troublesome eventually. It may, however, be beneficially employed in tic douloureux, and in parts on which a cicatrix would be objectionable. The moxa should be holden steadily, and as close as possible to the skin without touching, and until that skin becomes white from the detachment of the cuticle, and the formation of a blister. In a large proportion of cases, the superficial eschar, or *third* form, will be the best. To produce this eschar, the moxa must be placed on the skin, and kept there until that skin appears brown under it, and which will, in general, occur when the combustion has extended to the distance of a line from the spot. The *fourth*, or deep eschar, will be required when the disease is far from the surface, as in affections of the spinal marrow and hip. To perfect this eschar, the moxa must remain until its combustion is complete, and then the part will be black, and the surrounding skin slightly red, and wrinkled. Here it will sometimes be useful to increase the intensity of the heat by the blow-pipe, and, previously, the moxa ought to be surrounded by a cylinder of card-paper. Moreover, the spot should be marked with a little circle of ink in which the moxa is placed; and in using the blow-pipe, the adjacent parts must be protected by a dry piece of paper, antecedently wet in a saturated solution of the sulphate of alum, or muriate of soda, having a hole in its centre for the moxa. These solutions diminish the combustibility of the paper, and render a spark from the blow-pipe powerless. In paralysis, some obstinate forms of sciatica, and analogous cases, the frequent repetition of the moxa will be demanded. When the eschars are thrown off, ulcers are formed; and, when numerous, considerable irritation is excited, and a weak habit may be injured by their discharge. In these instances of deep-seated disease, the moxa, in conjunction with the needle, which is the *fifth* mode of application, has an excellent effect.

"When the moxa and acupuncture needle are used together, I perforate a moxa of a proper size, by a needle of such a length as will be sufficient to reach the seat of disease, and at the same time extend so far beyond the surface of the skin, as to keep the moxa about one inch from it, or so far as to secure the texture of the skin from injury. The needle is then introduced, as far as the seat of disease, by the assistance of the port-aiguille; and as soon as it has been introduced, the port-aiguille is removed, the needle being left in the part. The moxa, which had been previously perforated, should be now placed in a state of combustion on that end of the needle which projects beyond the surface of the skin, and allowed to burn round the needle by which it is thus transfixed. The heat disengaged from the moxa is communicated to the needle, and thence conveyed to the seat of disease. When the needle has cooled it is removed, and the wound or eschar produced by it is scarcely observable." 71.

Immediately after the application of the moxa, Baron Larrey advises us to wet the skin with the water of ammonia for the express object of preventing inflammation:—the very effect which those, that are ignorant of the true principle of the action of this agent, strive to accomplish. The aqua-ammoniæ succeeds in its purpose, and so do alcohol, æther, turpentine, ammonia, or even bruised garlic. In the first and last modes of employing the moxa no after treatment is needed. When eschars exist, they should be covered with adhesive plaster until they separate, which may be expected in eight, ten, and, occasionally, twenty days. The moxa acts more favourably when the eschar is thrown off very slowly. Superficial ulcers and excoriations may be washed once or twice daily with a solution of the nitrate of silver, or sulphate of copper, and covered by adhesive plaster.

Our analysis now brings us to the adjuvants of the moxa, since, like every other therapeutic agent, it is not above collateral aid. As those diseases, which are suited to the action of this remedy, consist in an atony, and often in an overloaded state of the absorbents and capillaries, the means, as adjuvants, may be placed under two divisions: 1st, Those that act by directly increasing the tone of the vessels, and consequently the rapidity of absorption and of capillary circulation; and,

2dly, Those whose beneficial influence depend on their emptying the overloaded or distended capillaries. The former are denominated tonics; the latter evacuants. In general, the mineral are preferable to vegetable tonics, especially mercury, iron, arsenic, silver, and copper. The only useful vegetable ones are cinchona, guaiacum, and turpentine. The precise doses of tonic medicines deserve consideration, and in numerous instances they may be greatly increased, and the medicines themselves occasionally changed and alternated. Local, like general tonics, are, essentially, stimulants, and produce their influence on the functions of capillary circulation and absorption, by exciting a degree of increased action, which is favourable to the acquisition of power. Those that can co-operate with the moxa, act either directly on the immediate seat of disease, or influence it by their action on the skin over the affected part. To the first class belong acupuncture, and probably galvanism; and to the second, the alternate aspersion of hot and cold water, trebinthinate and ammoniacal liniments, with other cutaneous stimulants. The acupuncture certainly acts as a stimulant, but whether by its mechanical power, or the disengagement of a minute portion of galvanic fluid, is doubtful. The operation of the moxa and acupuncture has already been explained, and the latter may also be used in conjunction with galvanism. To excite the capillary vessels to increased contraction, no remedies are more effectual than turpentine, ammonia, ice, or hot and cold water. Among those means that act on the general surface of the skin, hot baths, fumigations, &c. should be mentioned, and both may be either simple or medicated. Of those which act on a limited portion of the skin, the *vapour douche*, dry cupping, the long continued application of irritating embrocations, liniments, &c. must not be forgotten; nor blisters, scarifications, leeches, and those remedies that produce artificial eruptions, although all the latter act both as evacuants and counter-irritants.

The utility of pressure in disease needs no illustration. Since the days of Hippocrates and Celsus, friction has been a practice, more or less generally adopted, to relieve parts, in a state of debility, by mechanically assisting the too languid circulation of the fluids. Mr. Grosvenor, of Oxford, extended this practice considerably, but claims to originality he has none, as the reference to Celsus,* the mere echo of the words of Hippocrates, will decisively establish.

"Nor is this one art easily acquired by nurses of ordinary intelligence. If at any time I have occasion to increase the number of those rubbers, whom I keep in constant employment, I find that, with all the attention I can give to directions, my wishes are never carried into execution with accuracy. In fact, although it is a very mechanical process, it requires months of practice to render a person perfect in the art of employing it. I am convinced that the principal cause of the neglect of friction among the moderns, arises from the difficulty of obtaining persons properly experienced in the art. The imperfect manner in which the operation is commonly performed struck me forcibly some years ago, when, on a visit at Oxford, during the life of Mr. Grosvenor, I had an opportunity of observing the adroitness with which his rubbers used friction, and the steadiness with which they continued it for hours without any interruption. It was this visit which induced me to edu-

* De Medicina, Lib. II. sect. xiv. p. 76.

cate some poor persons in the art, and to keep them in my constant employment as rubbers." 93.

The bare hand, or the hand covered with a woollen glove, is the best instrument for friction. A small quantity of hair powder, or fine flour, should be interposed between the skin and hand to prevent any chafing. Great advantage will be derived by causing a gentle stream of vapour to play upon the part while friction is applied.

The various mechanical operations which have been denominated massing, shampooing, thumbing, kneading, &c. &c. may be included under the term percussion, since each and all of them essentially consist in the application of a circumscribed, momentary, or transient pressure, frequently repeated; and produce their beneficial effects by compressing the vessels to such a degree, as to increase the rapidity of their circulation. They may, perhaps, also act by a stimulating agency. These practices, although little used here, are much in vogue elsewhere, and especially in Finland, Russia, Turkey, Egypt, India, China, and the South Sea Islands, where they are universal, although the manner differs in different places. The hand is the best and most commonly employed mean for performing these various processes. It may be used in different ways—as by gently pressing or kneading with the thumbs; or with the ends of the fingers; or with the closed hand; or by causing the hand, or points of the fingers, to be propelled with an impulse upon the part; or, lastly, by grasping alternately, with either hand, the limb affected, one hand being placed at either side, in such a way that each may meet and surround the limb. But in every mode, and in every instance, the operation should be conjoined, whenever practicable, with the exposure of the patient, during the process, to the influence of heated air or vapour.

"If practice be necessary to enable an attendant to perform friction with art, it is still more necessary in shampooing. But the nurse-tender, who has been instructed and who has acquired a knowledge of friction, soon becomes an expert shampooer. Indeed, shampooing and friction may, and should, almost always be combined. The following is the manner in which these processes are performed at the Skin Institution. The patient, previously undressed, enters the shampooing apartment, already heated to the temperature of about 110° Fahr.* He then reposes on a cane sofa, and a small quantity of aqueous vapour, impregnated by passing through some aromatic or odoriferous herbs, is allowed to enter the apartment. This vapour gradually diffuses itself, and distributes a delightful fragrance. The patient remains in this situation until the surface of the body becomes gently moist. The shampooer now enters, and first submits the entire body of the patient to that degree of friction which may be necessary to disengage all branny or furfuraceous deposit from the surface of the skin. The process of shampooing is then commenced; and the shampooer, by gently grasping, pressing, and kneading with the fingers, thumb, or entire hand, variously modified and employed, from the extreme parts to the trunk, in the line of direction of absorption and venous circulation, continues the process for a longer or shorter time, according to the nature of the disease, and the feelings of the patient, the percussion being occasionally interrupted for the purpose of extending, in a gentle manner, such parts as are in a state of rigidity.

* "For a description of the shampooing apartments at the Dublin Skin Infirmary, see my account of the apparatus, &c. &c. illustrated by many plates. In 4to.

The whole process is then terminated by gently cleansing and drying the entire surface with a soft towel. The patient then withdraws into an adjoining apartment, the temperature of which is not inferior, when he enters it, to that of the shampooing room; but after he has reclined for some time on a bed or sofa, or after he has got himself dressed, the heat of this second room is gradually lowered to that of the external atmosphere, by a particular contrivance, but in so slow a manner, that no vicissitude whatever is experienced by the feelings of the patient. The beneficial consequences which always result from the process of shampooing, when properly employed, and in appropriate cases, are most striking. I have often known patients sink into a slumber, and enjoy several hours of delightful tranquillity after the operation, who had scarcely slept for weeks before; and I have known patients, who were scarcely able to move a limb on entering the apartment, and who were consequently carried into it, able to walk out erect, and with steadiness. Again, on the other hand, I have found a single operation produce feverish symptoms, and a restless night, when employed contrary to my wishes. The principles which have been laid down fully explain these opposite results. In fact, if those remedies, which are calculated to excite tone, be employed in the state of increased action, they will uniformly increase the disease, and aggravate every symptom, while the same cases will be benefited by opposite measures." 96.

It may be farther observed, that if remedies, which are adequate to diminish action, be persisted in beyond a proper time, they will render the disease more malignant in its nature, and more difficult of cure. "That disease," remarks, Mr. John Bell,* "which, with but a little indulgence, a very little encouragement of fomentations, poultices, bleeding, and low diet, would end in white swelling of the knee, may be stopped even by so simple a matter as a well-rolled bandage."

The work concludes with an account of thirty cases, which occurred in the private and hospital practice of Mr. Wallace, and were cured by different applications of the moxa. These cases may be briefly enumerated:—Nine of sciatica, and painful affections of the nerves; seven of lumbago, and other painful disorders of the muscles; eleven of paraplegia, and other paralytic affections; and three of epileptic, or spasmodic. Presuming, as we do most sincerely, on the accuracy and veracity of the reporter, and taking into consideration, as we ought to do, the unbiassed testimonies of other surgeons, we cannot help thinking that the efficacy of the moxa, as a therapeutic agent, in certain forms of disease, is incontestably established. A plate is annexed, which represents the porte-aiguille, the instrument for the application of evaporating lotions; the moxa forceps, with a moxa, surrounded by a bit of flat silver wire; a scarificator, of the form used by Baron Larrey; a blow-pipe, and a glass tube.

We recommend this work to every practitioner who is, and surely all are, anxious to alleviate human suffering, and desirous to understand the various modes of applying the moxa, and the true principles on which it is supposed to act. The author is an active and ingenious surgeon; he has had opportunities, and he has not neglected them: like every man that reasons, he is sometimes right and sometimes wrong; yet his facts are valuable. He is already favourably known to the professien; and if our approbation can impart pleasure, it is bestowed willingly, because deservedly.

* Principles of Surgery, vol i. p. 127.

